# Vascular stiffening and endothelial dysfunction in atherosclerosis

**DOI:** 10.1097/MOL.0000000000000852

**Published:** 2022-10-21

**Authors:** Aukie Hooglugt, Olivia Klatt, Stephan Huveneers

**Affiliations:** a Amsterdam UMC, University of Amsterdam, Department of Medical Biochemistry, Amsterdam Cardiovascular Sciences; b Amsterdam UMC, VU University Medical Center, Department of Physiology, Amsterdam Cardiovascular Sciences, Amsterdam, The Netherlands

**Keywords:** Aging, atherosclerosis, endothelium, mechanotransduction, stiffness

## Abstract

Aging is an important risk factor for cardiovascular disease and is associated with increased vessel wall stiffness. Pathophysiological stiffening, notably in arteries, disturbs the integrity of the vascular endothelium and promotes permeability and transmigration of immune cells, thereby driving the development of atherosclerosis and related vascular diseases. Effective therapeutic strategies for arterial stiffening are still lacking.

**Recent findings:**

Here, we overview the literature on age-related arterial stiffening, from patient-derived data to preclinical in-vivo and in-vitro findings. First, we overview the common techniques that are used to measure stiffness and discuss the observed stiffness values in atherosclerosis and aging. Next, the endothelial response to stiffening and possibilities to attenuate this response are discussed.

**Summary:**

Future research that will define the endothelial contribution to stiffness-related cardiovascular disease may provide new targets for intervention to restore endothelial function in atherosclerosis and complement the use of currently applied lipid-lowering, antihypertensive, and anti-inflammatory drugs.

## INTRODUCTION

The risk of developing cardiovascular disease is higher upon older age, with a majority of cardiovascular disease-related deaths occurring at an age of 70 years or older [1,2]. The high prevalence of cardiovascular disease in the elderly emphasizes the urge to understand vascular aging. Currently applied interventions for cardiovascular patients, such as antihypertensive ACE-inhibitors, statins, or anti-inflammatory agents are known to reduce cardiovascular risk significantly. However, these therapies do not suffice to prevent cardiovascular events in all patients and are not free from adverse effects. Targeting of the vascular response to age-related stiffening may serve as promising complementary approach of existing therapeutics, in particular for atherosclerotic lesions in the arterial wall.

The onset of atherosclerosis often occurs asymptomatically and it is difficult to predict the stage of atherosclerotic disease based on current traditional cardiovascular disease risk factors. Hypertension is a key cardiovascular risk factor, which precedes clinically identifiable atherosclerosis. Arteries undergo structural and functional changes upon aging and one of the main alterations is wall stiffening [3,4]. Arterial stiffening also precedes the development of cardiovascular disease and is emerging as an independent risk factor with predictive value for clinical outcome [3–5]. The predictive value of adverse cardiovascular events based on arterial stiffness is similar to other important risk factors such as left ventricular hypertrophy [6–8]. Arterial stiffening is a multifactorial process, which is caused by the increase in the deposition and crosslinking of extracellular matrix (ECM) proteins in the vessel wall. This process is accompanied by an increase in vessel wall thickness and artery size. Pulse wave velocity (PWV) is the standard macroscopic measurement of arterial stiffness in clinical diagnosis [9–11]. Indeed, PWV measurements in participants of the Framingham offspring cohort confirmed that vascular stiffening precedes the occurrence of hypertension and cardiovascular events [12,13]. This suggests that in patients with similar blood pressure for instance, the monitoring of differences in arterial stiffness helps to better predict the clinical outcome [14,15].

Currently applied therapeutic interventions already significantly reduce cardiovascular risk. Targeting the vascular response to stiffening, if used as combination therapy, could further improve such existing treatments [16]. Research aimed at therapeutic targeting of arterial stiffening has first focused on reversing the physical stiffening and outward remodeling of arteries. This approach turned out difficult due to lack of specificity and potency of drugs and the redundancy of involved signaling pathways [16]. Another approach to improve vascular function is to alleviate the response of the vascular cells (endothelial cells, vascular smooth muscle cells, pericytes, and fibroblasts) to arterial wall stiffening. The endothelium in healthy vessels activates various mechanotransduction pathways that maintain tissue integrity during the rapid and strong changes in mechanical forces that derive from pulsatile blood flow and vessel wall contractions. A healthy endothelial monolayer adapts to these forces while preserving its vascular barrier function. Upon disturbance in forces, however, like at atheroprone bifurcations or in stiff arteries, the endothelium fails to adapt and the arterial wall becomes prone to lipid accumulation and the infiltration of inflammatory cells [17,18]. Upon pathological vascular stiffening the endothelial cells fail to appropriately mechano-respond and become dysfunctional as barrier tissue [19–21]. Endothelial permeability is an essential step in the development and progression of early atherosclerosis [21,22^▪▪^]. A feedback loop of increased vascular stiffness and endothelial dysfunction promotes atherogenesis and its targeting has the potential to restore overall vascular homeostasis and counteract arterial stiffening.

This review provides an overview of current findings on arterial stiffening, from clinical studies to preclinical in-vivo and in-vitro experiments. We will overview the common techniques that are used to measure arterial stiffness and discuss patterns, and discrepancies, in the observed pathological and age-related arterial stiffening values. Finally, the endothelial responses to arterial stiffening, and current therapeutic strategies to attenuate them, are briefly mentioned. As effective therapeutic strategies for arterial stiffening are still lacking, improved models to better study and understand the endothelial role in arterial stiffness-related cardiovascular diseases will pave the way for new potential therapeutic strategies. 

**Box 1:**
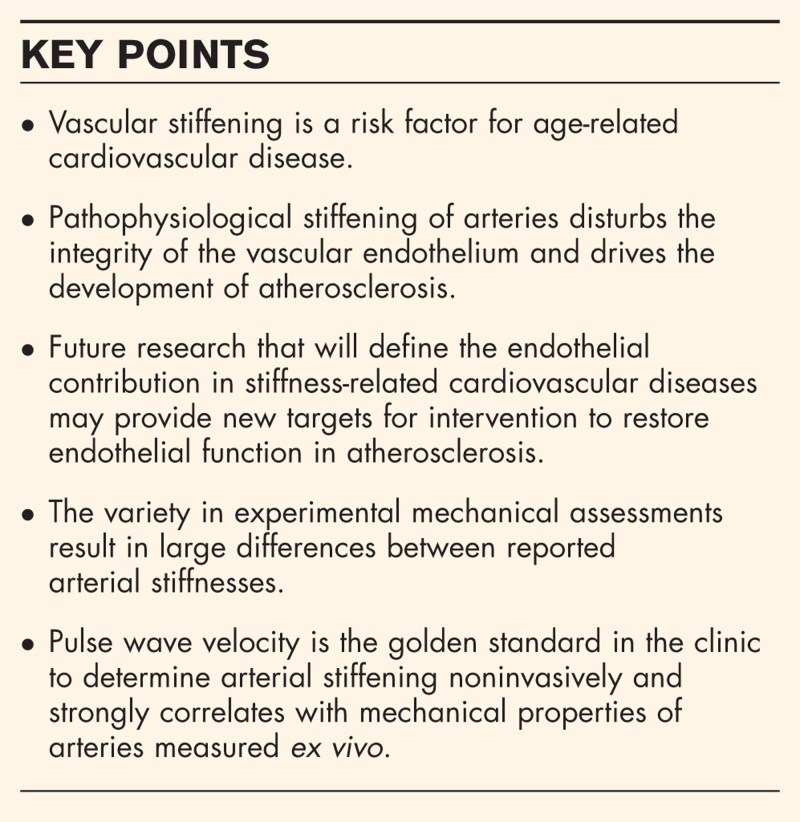
no caption available

## PATHOLOGICAL STIFFENING OF THE BLOOD VESSEL WALL

### Arterial wall layers and the extracellular matrix

Arteries are composed of three layers, namely the tunica intima, media, and adventitia layer. The intimal and medial layers, and the medial and adventitial layers are separated by the internal and external elastic membranes, respectively. The tunica intima consists of the endothelium, a monolayer of cells that lines the inner surface of the blood vessel, and a thin subendothelial layer of extracellular matrix, called the basement membrane. The basement membrane is composed of ECM proteins, such as laminins and collagen IV [23]. During aging and arterial stiffening, the relatively soft inner elastic membrane degrades and the intima thickens due to the activity of vascular smooth muscle cells (VSMCs) and the increased deposition of ECM proteins in the basement membrane [24,25].

The tunica media is comprised of multiple layers of VSMCs and ECM. Two important constituents of the ECM in the media are the elastin sheets and collagen fibers. Elastin contributes to the elastic properties of the blood vessel and its resilience to pulsatile pressure; whereas collagen fibers are not elastically deformable and determine the expansibility of the blood vessel [23]. VSMCs in the media are the effectors for blood vessel contraction, controlling vessel diameter, blood flow, and general arterial tone. VSMCs are also able to synthesize ECM proteins, in particular collagen I and III. Most VSMCs show a contractile phenotype, but during arterial stiffening VSMCs switch to a more proliferative and synthetic phenotype and increase their ECM production [26]. This increase in collagen fiber deposition, in combination with increased crosslinking of the ECM, increases the overall thickness and stiffness of the media during ageing. Endothelial cells undergo a similar process called endothelial-to-mesenchymal transition upon stiffening of their microenvironment, and consequently contribute to the stiffening of the intima layer by secreting fibrillar collagens, fibronectin and matrix metalloproteases that degrade laminin and elastin [27].

The tunica adventitia contains a variety of cell types, including immune cells and fibroblasts. The ECM of the adventitia consists of fibrillar collagens and proteoglycans, like versican, that contribute to the compressibility of the vessel wall. In healthy arteries, versican is abundant in the adventitia, but during atherogenesis versican expression is also expressed by VSMCs and endothelial cells and contributes to the pathological retention of lipids in the vessel wall [27]. Finally, in some cases, like in mature atherosclerotic plaques or end-stage renal disease, the intima or media undergoes calcification, respectively, which further contributes to arterial stiffening [26–28]. For in depth overviews of the remodeling of the vascular ECM during arterial aging and atherosclerosis, please read the following reviews [23,24,29,30].

### Measuring arterial stiffness in cardiovascular patients

The structural remodeling and stiffening of the arteries during aging is a strong prognostic indicator for cardiovascular risk, but it is not yet incorporated in routine clinical practice. One reason for this may be the wide variety of devices and methods that are being used to measure arterial stiffening. In this section we highlight pulse wave velocity (PWV), an ultrasound-based technique that is the golden standard in the clinic to assess arterial stiffness. The PWV is the speed of arterial pressure waves traveling along the arteries and is determined by taking the distance between two recording sites over the pulse transit time [31]. The pulse wave is typically measured at the carotid and femoral artery, as these sites provide reliable and reproducible measurements [32,33]. The brachial-ankle PWV is another established variant of PWV and has been reported to associate with cardiovascular risk factors to a similar extent [34]. However, in a retrospective study carotid-femoral PWV was a better predictor for heart failure [35].

The PWV provides an estimate of the average artery elasticity between the two recording sites. However, arterial stiffness is known to develop heterogeneously throughout the vascular system [36,37]. Ultrafast PWV is an upcoming technique that allows for detection of the local pulse wave at one site and is typically performed on the carotid artery, an area prone to atherosclerosis formation. This technique is not yet widely in practice in the clinic due to the lack of large population-based reference values of ultrafast PWV, but large cohort studies have demonstrated the reliability of this technique [38–41].

Upon a decrease in the elasticity of arteries (such as in old and stiff arteries), the propagation speed of the pulse wave increases resulting in higher PWV values. PWV values increase by age and is thus shown to be an independent predictor for cardiovascular risk. Current European guidelines point to a carotid-femoral PWV of more than 10 m/s as indicative of significant alterations of the vessel wall and high cardiovascular risk [32]. Clinical studies that demonstrate the association between PWV and cardiovascular disease have been previously reviewed [42^▪^].

In clinical practice, arterial stiffness is effectively synonymous to PWV. PWV levels are inversely related to the distensibility of arteries. The distensibility of an artery represents the relative change in cross-sectional area in response to pressure [31]. The distensibility thus represents the functional stiffness of the artery, which not only includes the composition of the artery (soft tissue properties, like the Young's modulus), but also properties like arterial diameter, blood pressure, and vascular tone (i.e., stiffness due to contractility cells in vascular wall). In other words, PWV is a technique that does not only assesses the structural remodeling and stiffening of the ECM during atherosclerosis. To measure the soft material properties of arteries, current techniques only allow for ex-vivo experimentation, where parts of the arteries are obtained from resected tissue of patients.

### Ex-vivo mechanical measurements of vascular stiffening

#### Bulk mechanical tests of arterial stiffness

To study the soft material properties of arteries in the context of vascular stiffening during aging and the progression of atherosclerosis, a variety of mechanical tests can be used. The principle underlying these techniques is that a certain force is applied to the artery (compression or extension force) and subsequently its deformation is monitored. Depending on the technique, material properties can be examined from macroscale to the nanoscale, providing insight from bulk mechanics of the artery to layer-specific mechanical properties.

The mechanical properties of atherosclerotic tissue has been investigated for quite some time. The first study that directly measured the mechanical properties of atherosclerotic lesions was reported in 1966 [43]. Since then the bulk mechanical properties of arteries and plaques have been investigated by a wide variety of techniques [44]. These techniques can be categorized by their type of deformation, e.g., arterial inflation, compression or extension, and the direction of the applied force (radial, circumferential, or longitudinal)[45]. Uniaxial tensile testing has been a frequently applied technique to characterize atherosclerotic tissues and provides insights on the forces that atherosclerotic plaques are exposed to during in-vivo rupture. Of note, there is a large variability in the reported data, which limits the extent to which mechanical properties of plaques can be compared between studies. Such differences between uniaxial tensile studies on atherosclerotic plaques have been acknowledged, implicating that a more standardized protocol for tissue preparation, tissue handling, mechanical testing, and postprocessing of data is needed [46].

Here, we provide a summary of uniaxial tensile studies that have reported the ultimate tensile stress of human atherosclerotic plaques. The ultimate tensile strength represents the maximum stress the artery can withstand while being stretched before rupture of the tissue. Two studies that investigated human carotid arteries have reported similar rupture stresses of noncalcified plaques to be around 342 ± 160 kPa [47] and 366.6 ± 220.5 kPa [48]. Measurements on a small set of diverse human aortic plaques showed a comparable ultimate tensile strength of 484 ± 216 kPa [49]. Calcification of human atherosclerotic plaques further increases the stiffness of the plaque but does not directly increase the risk of plaque rupture [47,48,50]. Larger calcifications are correlated with a reduced risk in plaque rupture and are thought to stabilize the plaque, whereas superficial or spotty calcification increase the risk [51]. In the latter case, calcification is thought to make the plaque more susceptible to rupture by increasing the asymmetry in mechanical stresses within the plaque.

To obtain more insight in the differential stresses inside the plaque, researchers have separated the different layers of arteries (and plaques) before tensile testing. Characterization of the mechanical properties of different layers not only provides insight in the relative stiffness between each layer but could also help with modelling plaque rupture *in situ*[52,53]. Here, we overviewed studies that have performed biaxial tensile testing on separated layers of human arteries. Independent of vascular beds, the tunica adventitia has a much higher tensile strength than the media or the intima (Table 1). The adventitia is known for its role to dampen and dissipate energy from the pulsatile blood flow [54], which is likely reflected by the high tensile strength values. From these studies, it is not possible to provide a general conclusion on differences between the media or the intima, as the ultimate tensile strength is too variable. This variability could be explained by the different origins of the vascular bed, the stage of atherosclerosis, and/or the measured axis. Interestingly, in atherosclerotic carotid arteries, the media in the circumferential axis was stiffer compared to the longitudinal axis, but no differences were observed within the intima or adventitia (Table 1). The differences in tensile strength between the circumferential and longitudinal axes are likely attributed to the specific composition of the artery walls, which potentially also plays a role in the distinct susceptibility to plaque formation in different vascular beds. Interestingly, the intima of the iliac artery has a lower tensile strength in the atherosclerotic region as compared to nondiseased regions (Table 1), which reflects the fragility of the fibrous cap and the potential increased risk of rupture [56].

**Table 1:** Ultimate tensile stress of the intima, media, and adventitia of human arteries from different vascular beds, tested in the circumferential and longitudinal direction (ultimate tensile stress in kPa, mean values ± SD)

		Ultimate tensile strength (kPa) per arterial layer			
Vessel	Condition	Intima	Media	Adventitia	Ref.
***Measurements along circumferential axis***					
Iliac	Nondiseased	488.6 ± 185.6	202.0 ± 69.8	1031.6 ± 306.8	[56]
	Athero	254.8 ± 79.8	1073.6 ± 289.3	–	
Coronary	Nondiseased	394.0 ± 223.0	446.0 ± 194.0	1430.0 ± 604.0	[55]
Carotid	Athero	1022 ± 427	1230 ± 533	1802 ± 703	[57]
***Measurements along longitudinal axis***					
Iliac	Nondiseased	943.7 ± 272.3	188.8 ± 110.9	951.8 ± 209.0	[56]
	Athero	468.6 ± 100.1	187.4 ± 8.3	–	
Coronary	Nondiseased	391.0 ± 144.0	419.0 ± 188.0	1300.0 ± 692.0	[55]
Carotid	Athero	1047 ± 536	519 ± 270	1996 ± 867	[57]

#### Atomic force microscopy-based assessment of local arterial stiffness

While bulk mechanical testing is an established technique, the development of more accurate and precise tools has allowed the probing of mechanical properties of the vascular microenvironment in more detail to unravel the development of arterial stiffening on local cellular level. In particular, atomic force microscopy (AFM) is a valuable technique to assess the local topography and mechanical properties of tissues *ex vivo*, reaching a higher precision compared to for instance micro- and nano-indentation approaches. AFM uses a cantilever to indent the tissue to determine its local stiffness. In the last decade, AFM has become a broadly used method to study the stiffness of vascular cells and tissues both *in vitro* and *ex vivo*[58]. Next, we overviewed AFM studies that have assessed the mechanical changes of the vessel wall within the context of cardiovascular disease and aging (Table 2). Table 2 presents information on the type of tissue investigated and its associated elasticity, but also reports the applied probing direction and the geometry of the AFM tip. "Cross section” indicates a sagittal ring slice of the vessel, which allows for probing of the different layers of the artery. "En face” is when the vessel is opened longitudinally into a flat slice, which allows for reliable probing of the thin intimal layer from the luminal side [59]. Some studies opted for removing the endothelium when probing en face, to directly characterize the stiffness of the underlying basement membrane [21,60,61]. The relationship between the stiffness of the basement membrane and the endothelium is not well studied. One study directly compared the stiffness of intact and endothelial denuded bovine carotid arteries en face and found comparable stiffness values (2.5 kPa and 2.7 kPa, respectively [62]). Table 2 also includes the geometry of the used AFM probes, as this parameter has a pronounced effect on the reported stiffness of vascular tissue. For instance, a drastic change was observed in measured stiffness values of pulmonary vessels when the probe was changed from a pyramidal to a spherical shape (from 67.66 kPa to 8.36 kPa, respectively) [63] (Table 2). Taken together, the reported Young's modulus varies greatly between studies, with the lowest reported stiffness of 0.46 kPa for mouse aortic endothelium [64^▪^] and the highest stiffness of 2500 kPa for elastin fibers in the aorta of old mice [65^▪^] (Table 2). This variability in reported stiffness moduli cannot only be explained by differences in the AFM tip geometry and requires a close comparison of experimental conditions between studies, which is beyond the scope of this review.

**Table 2:** Summary of AFM studies investigating the stiffness of arteries during the progression of cardiovascular disease and aging

Vessel	Species	Age	Tissue	Condition	Young's modulus (kPa)	Probing	AFM tip (shape, radius)	Ref.
***Atherosclerosis/ hypertension/ high-fat, high sucrose diet studies***								
Coronary artery	Human	39.3 ± 5.1 years	Media	No plaque	10.7 ± 3.3	Cross-section	Conical	[67^▪▪^]
		41.6 ± 3.8 years		Early plaque	12.1 ± 3.1			
		45 ± 5.9 years		Advanced plaque	15.5 ± 3.6			
		41.6 ± 3.8 years	Intima (fibrotic)	Early plaque	8.7 ± 1.5			
		45 ± 5.9 years		Advanced plaque	17.6 ± 3.2			
Aorta	Mouse, ApoE-/-	25–30 weeks	Intima	Lipid rich area	5.5 ± 3.5	Cross-section	Spherical, 5 μm or 12 μm	[68]
				Fibrotic, SMC rich	10.4 ± 5.7			
				Fibrotic, hypocellular	59.4 ± 47.4			
Aorta	Mouse, ApoE-/-	42–56 weeks	Media	Control	9.8	Cross-section	Spherical, 5 μm	[70]
			Lipid core	Atherosclerosis	1.5			
Aorta	Mouse, ApoE/LDLR-/-	6 months	Endothelium	Control	0.46 ± 0.023	En face	Spherical, 10 μm	[64^▪^]
				Atherosclerosis	0.56 ± 0.022			
Aorta	Mouse, ApoE-/-	6 months	Endothelium	Control	4.8^a^	En face	Spherical, 1 μm	[69]
				Atherosclerosis	15.4^a^			
Pulmonary arterioles (diameter<100 μm)	Human	18–71 years	Lung tissue	Control	2.0^a^	Cross-section	Spherical, 2.5 μm	[73]
				PAH	3.6^a^			
Ascending aorta	Mouse, C57BL/6	10–11 months	Endothelial denuded vessel	Normal diet	24 ± 2.8	En face	Spherical, 5 μm	[61]
				High fat, high sucrose diet	52 ± 4.8			

***Aging studies***								
Pulmonary arterioles (diameter<100 μm)	Human	11–30 years	Lung tissue	Young	5.06 ± 2.43 kPa	Cross-section	Spherical, 2.5 μm	[63]
		41–60 years		Old	9.09 ± 4.28 kPa			
Thoracic aorta	Mouse, C57BL/6	10–11 weeks	Endothelial denuded vessel	Young	31.9 ± 4.5	En face	Spherical, 10 μm	[21]
		20–25 months		Old	70.6 ± 8.8			
Thoracic aorta	Mouse, C57BL/6	2 months	Endothelial denuded vessel	Young	24.7^a^	En face	Spherical, 5 μm	[24]
		18 months		Old	32.9^a^			
Thoracic aorta	Mouse, C57BL/6	2 months	Endothelium	Young	3.1^a^	En face	Spherical, 1 μm	[76]
		6 months		Middle aged	3.6^a^			
		12 months		Middle aged	16.9^a^			
		18 months		Old	21.8^a^			
Thoracic aorta	Mouse, C57BL/6	12 weeks	Endothelium	Young	1.3^a^	En face	Conical tip	[77^▪^]
		86 weeks		Old	1.9^a^			
Ascending aorta	Mouse, C57BL/6	0.5 months	Intima	Young	2.8^a^	En face	Spherical, 15 μm	[75]
		2.0 months		Middle aged	5.1^a^			
		3.5 months		Middle aged	4.4^a^			
		0.5 months	Media	Young	12.8^a^			
		2.0 months		Middle aged	38.7^a^			
		3.5 months		Middle aged	36.6^a^			
Pulmonary artery	Bovine	3 weeks (neonatal)	Intima	Young	0.9^a^	Cross-section	Spherical, 5 μm	[74^▪^]
		20–30 months	Intima	Old	2.5^a^			
		3 weeks (neonatal)	Media 1	Young	0.9^a^			
		20–30 months	Media 1	Old	1.5^a^			
		3 weeks (neonatal)	Media 2	Young	1.5^a^			
		20–30 months	Media 2	Old	2.5^a^			
		3 weeks (neonatal)	Adventitia	Young	1.4^a^			
		20–30 months	Adventitia	Old	1.3^a^			
Aorta	Mouse, C57BL/6	1 month	Elastin fibers between SMCs	Young	90 ± 20	Cross-section	Pyramid	[65^▪^]
		5 months		Middle aged	510 ± 100		Conical	
		10 months		Middle aged	1200 ± 330		Conical	
		20 months		Old	2500 ± 260		Conical	

a In cases where no exact stiffness values were reported, values were estimated from graphs. These values are marked in the table with an ^a^. AFM, atomic force microscopy, SMC, smooth muscle cell.

The first study to characterize human plaques by nanoindentation, used a probe tip with a diameter of 200 μm and indentation depth of 5 μm, and reported Young's moduli of 270 ± 150 kPa for fibrous tissue, 2.1 ± 5.4 MPa for partially calcified fibrous tissue, and 0.7 ± 2.3 GPa for calcified tissue [66]. To our knowledge, a decade later, only one study has used AFM to elaborately examine the stiffness of the different layers of the human arterial wall and plaque components during atherosclerotic development [67^▪▪^]. This study categorized coronary arteries into healthy, mildly diseased, and advanced atherosclerotic plaques and investigated the elasticity of the internal and external elastic laminas, the media, and the plaque components during disease progression. The stiffness of the internal elastic lamina decreased (from 34.9 kPa to 24.8 kPa), while the stiffness of the external elastic lamina and the media did not change so much (from 34.2 kPa to 31.9 kPa) [67^▪▪^]. The media and intima layers increased in stiffness during plaque progression and have a comparable stiffness in the advanced plaque (Table 2). Within the atherosclerotic plaques, the lipid core was the least stiff area (2.7 ± 1.8 kPa), whereas the calcified areas were the most stiff (96.1 ± 18.8) [67^▪▪^].

Due to the limited availability of human vascular material, the ApoE and LDLR knockout atherosclerotic mouse models have been used to study arterial stiffening during atherosclerosis [64^▪^,^68^–^70^]. In AFM studies performed on mouse aortic atherosclerotic plaques, the lipid core was also the lowest reported elasticity, with reported Young's moduli of 5.5 kPa ± 3.5 [68] and 1.5kPa [70] and has a similar elasticity to the human lipid core. It is however hard to draw a general conclusion concerning the stiffness regulation within the arterial wall, as the examined plaque components and the order of magnitude of the Young's moduli varies drastically (Table 2). The atherosclerotic plaque of mice differs considerably in its biomechanical composition compared to human plaques and is considered resistant to plaque rupture [71]. The use of tissue-engineered plaques to study the mechanical properties of human plaque could be an interesting alternative [72^▪^].

To study the effect of aging on arterial stiffness, the stiffness of young and old blood vessels have been compared based on various AFM experiments (Table 2). There is however no clear consensus age cutoff that defines young or old human vessels in current literature. In a study investigating the stiffness of human pulmonary arterioles, tissues above 40 years old were considered old and these arterioles showed almost a twofold increase in stiffness [63]. Of note, arterial stiffening has not only been observed in large arteries, but also in aged human pulmonary arterioles and in pulmonary arterial hypertension [73,74^▪^]. In the mouse studies generally tissues above 20 months of age were considered old (Table 2). The earliest increase in arterial stiffness, measured by AFM en face, was already observed between aortas from 0.5 month old mice and 2 months old mice [75]. Interestingly, in this study there was no further increase observed in arterial stiffness between 2 and 3 months old mice. Similarly, another study found no increase in arterial stiffness of the aorta between 2 months old and 6 months old mice [76]. However, the arterial stiffness did drastically increase between 6 months old and 12 months old mice. These results indicate that arterial stiffness might increase during specific moments of the lifespan of mice (i.e., before 2 months of age, and from 6 months and older). By contrast, by applying cross-sectional AFM on mouse aorta, it was found that elastin fibers increase in stiffness between 1 month old and 5 months old mice [65^▪^], indicating potential differential regulation of ECM components during aging. In line with this, arterial stiffening was mainly observed in the intima and media, but not the adventitia in aging pulmonary arteries [74^▪^]. In this study, the Young's modulus of the intima, media, and adventitia of the aorta, carotid, and pulmonary arteries were compared. See Table 2 for the discussed experimental results. Interestingly, the Young's modulus was similar between the arterial layers, while the tensile strength of the adventitia has previously been reported to be significantly higher (see Table 1). The adventitia is perhaps not so stiff during compression or has a relatively low stiffness at small deformations (as applied during AFM). These observations show that different mechanical tests can provide complementary insights of the mechanical properties of aging arteries. In addition, some studies have compared AFM and PWV techniques to investigate the relationship between stiffness as a material property and functional stiffness *in vivo*; and a striking linear positive correlation was found between wave velocity and the Young's modulus [60,65^▪^].

### Stiffness-induced endothelial dysfunction

How changes in mechanical forces in the vascular microenvironment affect endothelial functions and play a prominent role in atherosclerosis development has been extensively overviewed before [78–80]. Vascular stiffening disturbs endothelial barrier function [20]. The stiffness of the vessel wall ECM is sensed on an endothelial cell level through integrin-based adhesions, which signal and transmit forces from ECM to the intracellular actomyosin cytoskeleton [81]. In turn, contraction of the actomyosin cytoskeleton exerts tension on VE-cadherin- and PECAM1-based endothelial cell-cell junctions [82–84]. The excessive stiffening of the vessel wall in aging arteries perturbs this mechanotransduction response and thereby weakens the endothelial cell-cell junctions [19,24], resulting in leaky endothelium, increased inflammation and driving the development of hypertension and atherosclerosis [20,24,85,86]. The vascular wall of the aorta and other arteries from ApoE-/- mice are stiffer than in wild type mice, which is caused by increased ECM deposition and crosslinking by lysyl oxidase, rather than their high plasma cholesterol levels [69]. The stiffening was partially reversible, as treatments with a lysyl oxidase inhibitor reduced arterial stiffness and atherosclerosis development. The importance of endothelial actomyosin activation for atherogenesis, downstream of stiffness sensing, is further demonstrated by experiments which showed that deficiency of myosin light-chain kinase, an important activator of actomyosin contraction, attenuates endothelial permeability in this atherosclerotic mouse model [87]. In addition, the inhibition of Rho kinase-mediated actomyosin contractility, improved endothelial barrier function in stiff aorta of aged mice [21]. ECM stiffness also activates the transcriptional co-activators YAP and TAZ in endothelial cells [88,89], which are important drivers of atherosclerosis development [90,91]. Interestingly, there is tight crosstalk between YAP/TAZ activation and endothelial metabolism, including the control of glycolysis, amino acid synthesis, lipid synthesis, and mitochondrial activity [92,93]. This may explain why age-related arterial stiffening is worsened by diabetes and metabolic syndromes in cardiovascular patients [94].

## CONCLUSION

Atherosclerosis remains a leading cause of mortality worldwide, despite being one of the research focuses for decades. With this review, we highlighted the knowledge concerning vascular stiffening and mention endothelial mechano-responses downstream of stiffness-sensing as a key process for therapeutic targeting to attenuate atherogenesis. Improving our knowledge regarding the changes in mechanical properties of the human arterial wall during aging and implementing them in in-vitro experimental setups that assess endothelial cell function, will be crucial to search for effective targeting strategies that inhibit the atherogenic endothelial contribution.

Statins are widely known to lower cholesterol levels by inhibiting 3-hydroxy-3-methylglutaryl coenzyme A, a key enzyme in cholesterol synthesis. In addition to its cholesterol-lowering effect, statins have attenuated arterial stiffness in vitro and in human clinical studies by alleviating stiffness-induced Rho-mediated endothelial mechano-responses [95,96]. Statins also suppress YAP/TAZ activity and thereby inhibit vascular inflammation [90]. Statin therapies have been in the clinic for many years and have proven to be safe and effective, to a certain extent, preventing cardiovascular events regardless of the patient's age [97]. Another promising therapeutic for the treatment of atherosclerosis is the RhoA/ROCK inhibitor fasudil. This drug improves endothelial function in experimental cardiovascular animal models [98,99]. However, a clinical study has yet to confirm this promising effect for patients. The clinical trial NCT02145468 investigated the effects of fasudil on carotid atherosclerosis but was terminated in 2017 due to poor enrollment [ClinicalTrials.gov identifier: NCT00670202].

It is important to note that nontherapeutical approaches have been investigated to reduce arterial stiffness. A healthy lifestyle has been widely shown to increase cardiovascular health [100]. In experimental settings, arterial stiffness was reduced when obese mice were switched to a normal diet, or when old mice were forced to physical exercise [59,60]. Finally, stiffening of arteries during aging raises the risk of cardiovascular disease in both men and women. Intriguingly, the vasculature is less stiff in young women compared to age-matched men, yet arterial stiffening is strongly elevated in aged women compared to men [101,102]. This indicates that there is an unequal increase in stiffening in postmenopausal women. It is therefore very important to consider sex an important variable in future cardiovascular research that explores age-related stiffening.

## Acknowledgements

*None.*

### Financial support and sponsorship

*This study was financially supported by the Dutch Heart Foundation (Dekker Established Investigator grant 03-002-2021-T087) and the Amsterdam Cardiovascular Sciences institute.*

### Conflicts of interest

*There are no conflicts of interest.*

## REFERENCES AND RECOMMENDED READING

Papers of particular interest, published within the annual period of review, have been highlighted as:

▪ of special interest
▪▪ of outstanding interest
